# Supplementary material for: Identification of transcription factor co-regulators that drive prostate cancer progression
Source: Sci Rep. 2020 Nov 23;10:20332. doi: 10.1038/s41598-020-77055-5 (PMC7683598; doi:10.1038/s41598-020-77055-5)
Supplement: Supplementary file 2 — Supplementary Figures. [file 41598_2020_77055_MOESM2_ESM.pdf]

Supplementary Data

Identification of transcription factor co-regulators that drive prostate cancer progression

Manjunath Siddappa[1], Sajad A. Wani[2], Mark D. Long[3], Damien A Leach[4], Ewy A. Mathé[5,6], Charlotte L. Bevan[7], Moray J. Campbell[1,8,9*].

[1] College of Pharmacy, Pharmaceutics and Pharmaceutical Chemistry, 536 Parks Hall, 500 West 12th Ave., The Ohio State University, Columbus, OH 43210. smanju712@gmail.com

[2] College of Pharmacy, Pharmaceutics and Pharmaceutical Chemistry, 536 Parks Hall, 500 West 12th Ave., The Ohio State University, Columbus, OH 43210. wani.16@osu.edu

[3] Department of Cancer Genetics and Genomics, Roswell Park Comprehensive Cancer Center (RPCCC), Buffalo, NY, 14263, USA. Mark.Long@RoswellPark.org

[4] Imperial Centre for Translational and Experimental Medicine, Department of Surgery & Cancer, Imperial College London, Hammersmith Hospital, Du Cane Road, London, W12 0NN, UK. damien.leach@imperial.ac.uk

[5] Biomedical Informatics Department, College of Medicine, The Ohio State University, Columbus, Ohio, 43210, USA.

[6] Division of Preclinical Innovation, National Center for Advancing Translational Sciences, NIH, 9800 Medical Center Dr., Rockville, MD, 20892, USA. ewy.mathe@nih.gov

[7] Imperial Centre for Translational and Experimental Medicine, Department of Surgery & Cancer, Imperial College London, Hammersmith Hospital, Du Cane Road, London, W12 0NN, UK. charlotte.bevan@imperial.ac.uk

[8,9] The James, Comprehensive Cancer Center, The Ohio State University, Columbus, Ohio, 43210, USA. Biomedical Informatics Shared Resource, The Ohio State University, Columbus, Ohio, 43210, USA. Campbell.1933@OSU.edu

[*]Corresponding author

Supplementary Figure Legends

Figure legends

Supp. Figure 1. Family-wide analyses of transcription factors (TFs), coactivators (COAS), corepressors (CORS) and mixed function coregulators (MIXED) in PCa cohorts. Bootstrapping permutation approach was used to test if observed family-wide changes were more than predicted by chance. The heatmap **(A)** shows CORS expression in the TCGA cohort, indicating the genes are commonly down-regulated. To test CORS down-regulation we identified the proportion of these genes altered by > 2 Z-scores (vertical dashed red line, **A, right panel**) and compared this to the proportion of all gene families down-regulated in sets of the same size (green histogram); a similar analyses was applied to the up-regulated genes (red histogram). The observed proportion of CORS down-regulated by > 2 Z-scores was significantly more (and hence, to the right) than the estimated proportion of down-regulated groups of the same size and randomly sampled ($p=1e^{-05}$). The CNA data and normalized exome mutation rate were treated in the same manner. Positive controls included gene groups known to be significantly altered by expression (nuclear hormone receptor (NR) down-regulation; HOX family (HOX)(61) up-regulation), mutation (Cosmic_mutant) (**B**)

Supp. Figure 2. Correlation of mRNA and protein expression of transcription factors (TFs), coactivators (COAS), corepressors (CORS) and mixed function coregulators (MIXED) in PCa cohorts. Correlation between RNA (TCGA or SU2C) and protein (OICR) when considering RNA transcripts for detected proteins.

Supp. Figure 3. Identification of commonly altered transcription factors (TFs), coactivators (COAS), corepressors (CORS) and mixed function coregulators (MIXED) in the OICR cohort. Proteins commonly altered in OICR cohorts were identified and visualized as heatmaps

Supp. Figure 4. Enrichment of transcription factors in LNCaP cells with stable knockdown of PGC1α. LISA cistrome analyses of the PGC1α-dependent genes revealed that the top 50 transcription factors associated with these DEGs

Supp. Figure 5. Gene set enrichment analyses of PGC1α dependent RNA-Seq. Top eight positive and negative significant (FDR < .1) normalized enrichment scores (NES) in four gene set categories; Hallmarks, Curated, GO and Reactome sets.

Supp. Figure 6. Cumulative correlation of PGC1α and target genes. Cumulative correlation plots revealed that the empirical correlation between the expression of *PPARGC1A* and genes regulated by *PPARGC1A* shRNA (green symbol, KS test; p<1e-9) and genes directly bound by PGC1α (purple symbol, KS test; p<1e-9).

Supp. Figure 7. Expression of PPARGC1A in prostate cancer cell lines. PPARGC1A in a PCa cell lines in the Broad Cancer Cell Line Encyclopedia

A

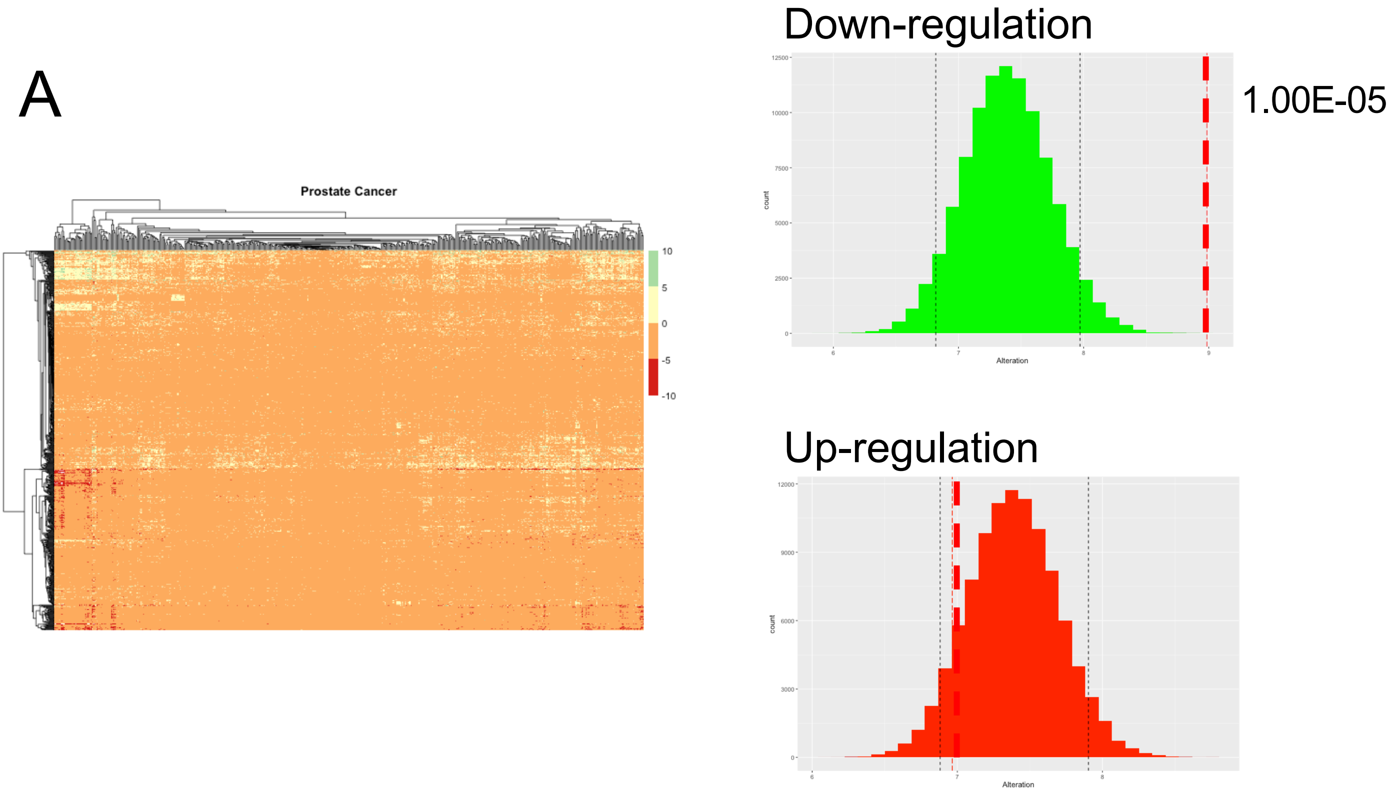

B

Group	center	Test	logPV	threshold
NR	TCGA	rank.up.L	1.978981	Significant
NR	MSKCC	rank.up.L	1.074692	Significant
Hox.nkl	TCGA	rank.up.H	3.193824	Significant
Hox.nkl	NEURO	rank.up.H	1.539870	Significant
HOX	FH	rank.up.H	1.510649	Significant
Hox.nkl	FH	rank.up.H	2.434157	Significant
Hox.nkl	TCGA	rank.down.L	3.795884	Significant
Hox.nkl	MSKCC	rank.down.L	3.795884	Significant
NR	NEURO	rank.down.L	3.795884	Significant
Hox.nkl	NEURO	rank.down.L	3.795884	Significant
HOX	FH	rank.down.L	2.079881	Significant
Hox.nkl	FH	rank.down.L	3.290734	Significant
NR	TCGA	rank.down.H	1.074692	Significant
NR	MSKCC	rank.down.H	2.892794	Significant

Group	center	Test	logPV	threshold
cosmic.mutants	TCGA	rank_cds_H	1.928122	Significant
cosmic.mutants	NEURO	rank_cds_H	1.928122	Significant
cosmic.mutants	SU2C	rank_cds_H	1.896885	Significant
cosmic.mutants	FH	rank_cds_H	3.698974	Significant
cosmic.mutants	MICH	rank_cds_H	1.928122	Significant

C

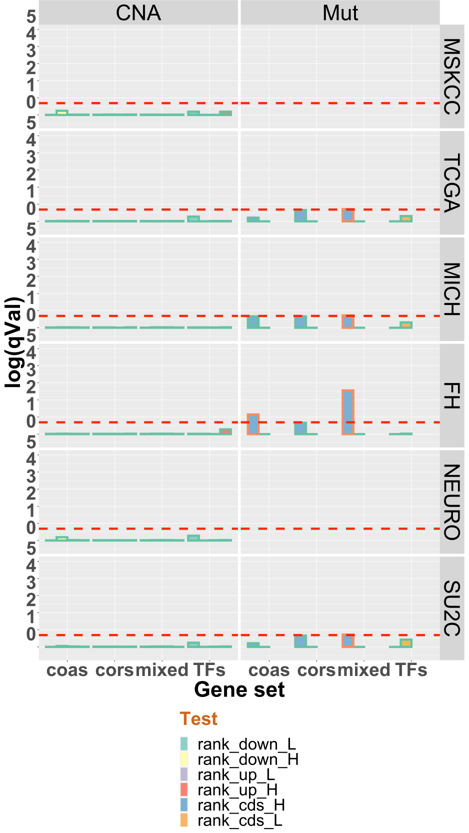

Supplementary Figure 1

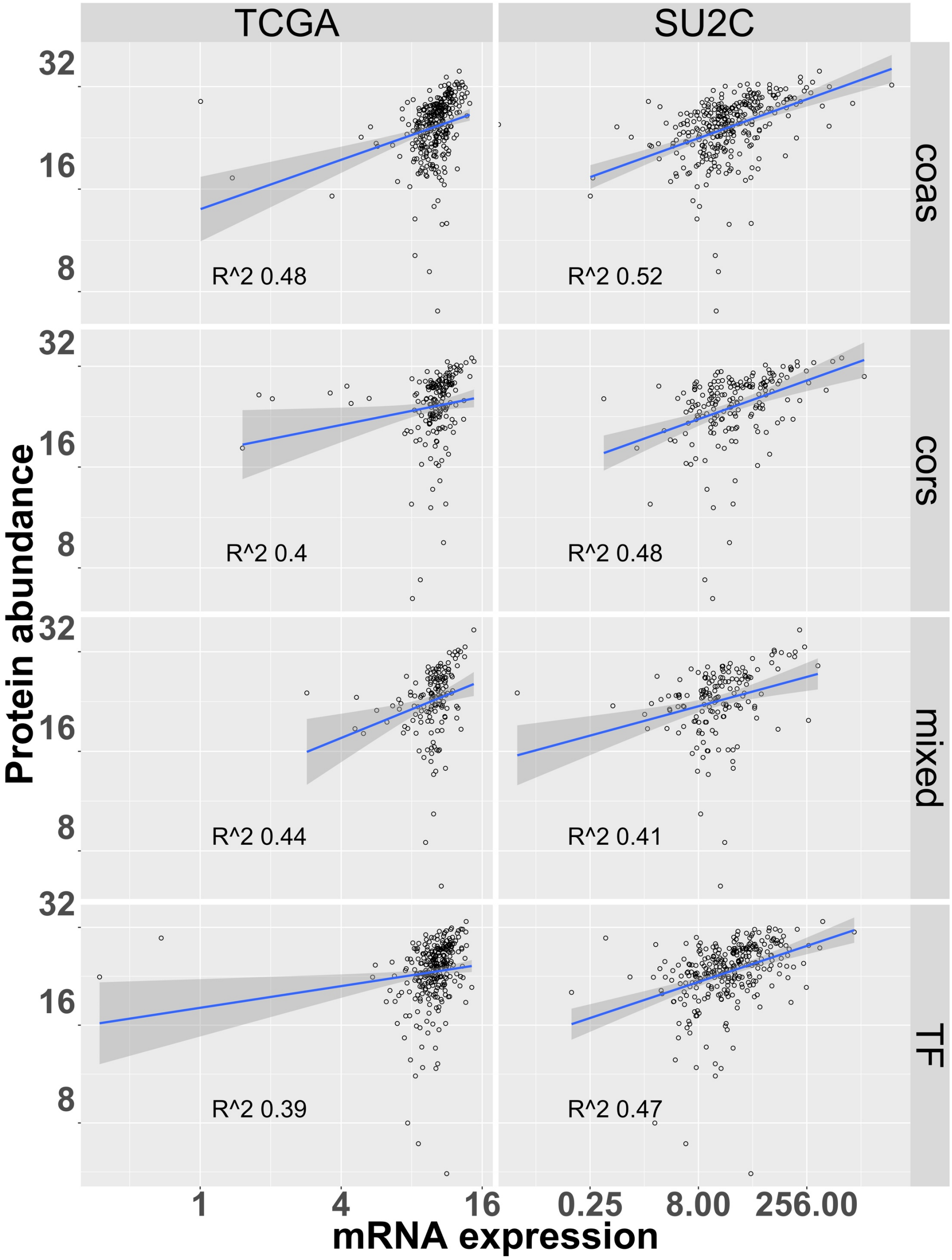

Supplementary Figure 2

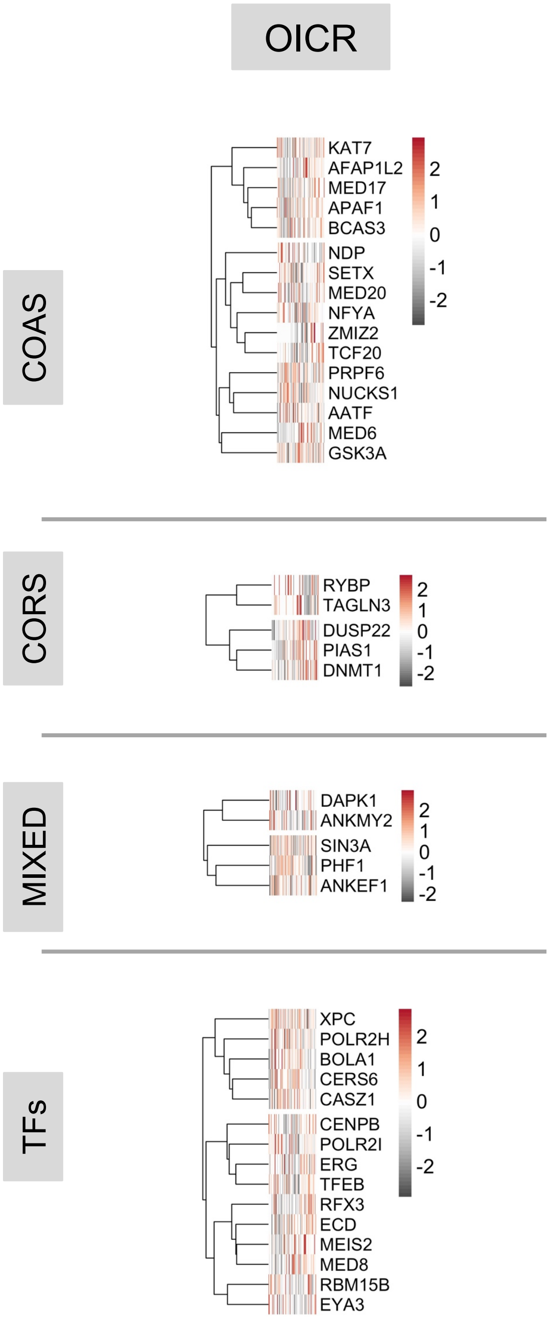

Supplementary Figure 3

Transcription.Factor	rank	logPV
FOXA1	1	42.045275
AR	2	41.221849
GATA2	3	30.764472
HOXB13	4	30.143271
PIAS1	5	29.229148
NR3C1	6	26.408935
CTRL	7	21.096367
FOXA2	8	19.420216
CEBPB	9	17.274905
GATA3	10	15.135489
NKX3−1	11	14.410050
LMNB1	12	14.238824
HDAC3	13	13.655608
TET2	14	13.389340
SOX4	15	12.995679
PRDM6	16	12.924453
PBX3	17	12.920819
NUP98−HOXA9	18	10.494850
LMNA	19	10.490797
ESR1	20	10.430626
BMI1	21	10.386158
SP140	22	10.343902
RNF2	23	10.130182
SUMO2	24	10.049635
HES2	25	9.966576
PR	26	9.600326
PSMA7	27	9.559091
SMARCA4	28	9.422508
FOS	29	9.208309
NANOG	30	9.111821
SOX2	31	8.869666
ERG	32	8.747147
MYC	33	8.425969
FOXH1	34	8.403403
ZBTB48	35	8.294136
CTCF	36	8.218245
PDX1	37	8.199283
AFF1	38	8.186419
SIX2	39	8.025949
GLI2	40	8.014125
LHX2	41	7.838632
SMC1A	42	7.804100
BATF3	43	7.777284
PPARG	44	7.739929
STAT3	45	7.705534
5MC	46	7.655608
GATA6	47	7.542118
WDR5	48	7.467246
HLF	49	7.255707
RELA	50	7.176526

Supplementary Figure 4

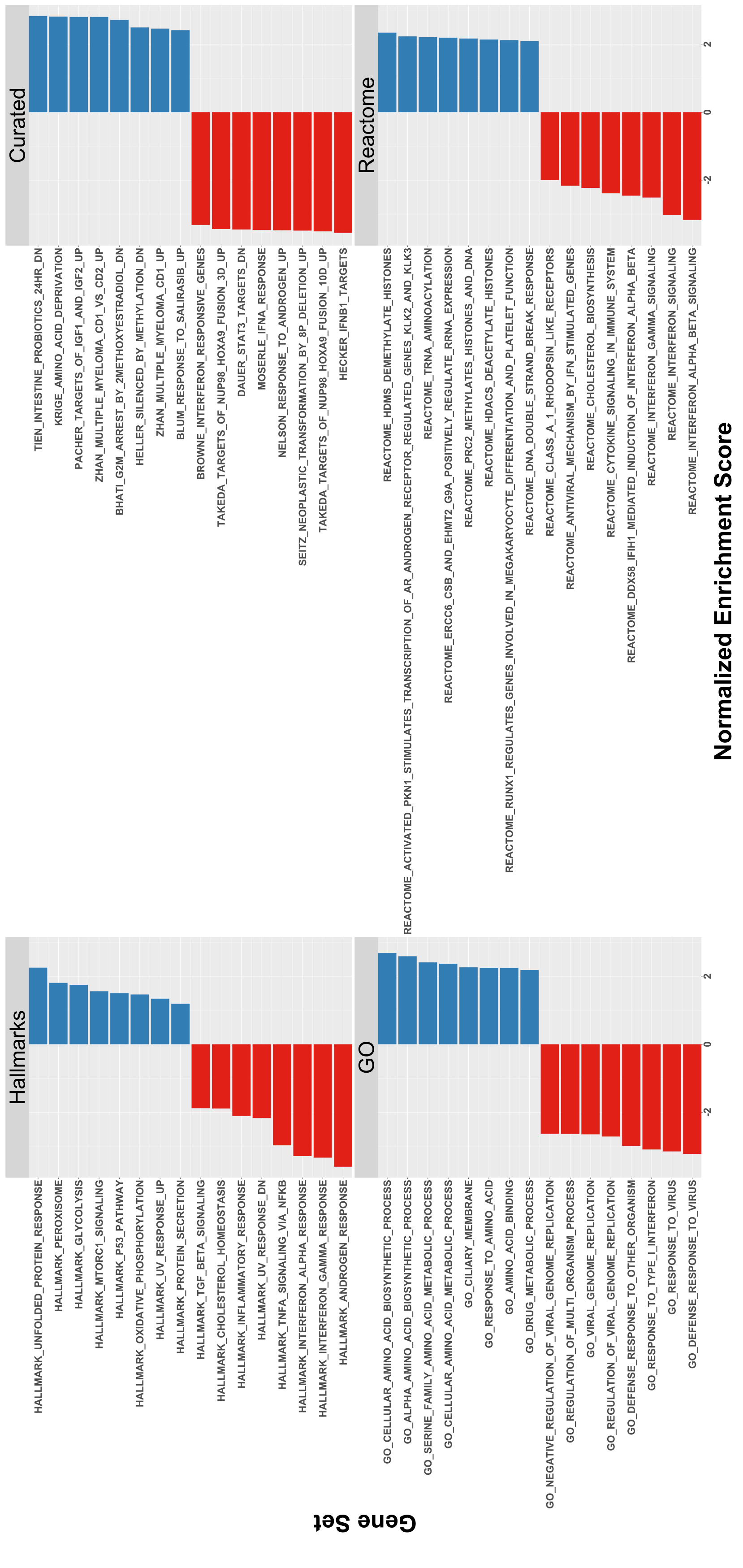

Supplementary Figure 5

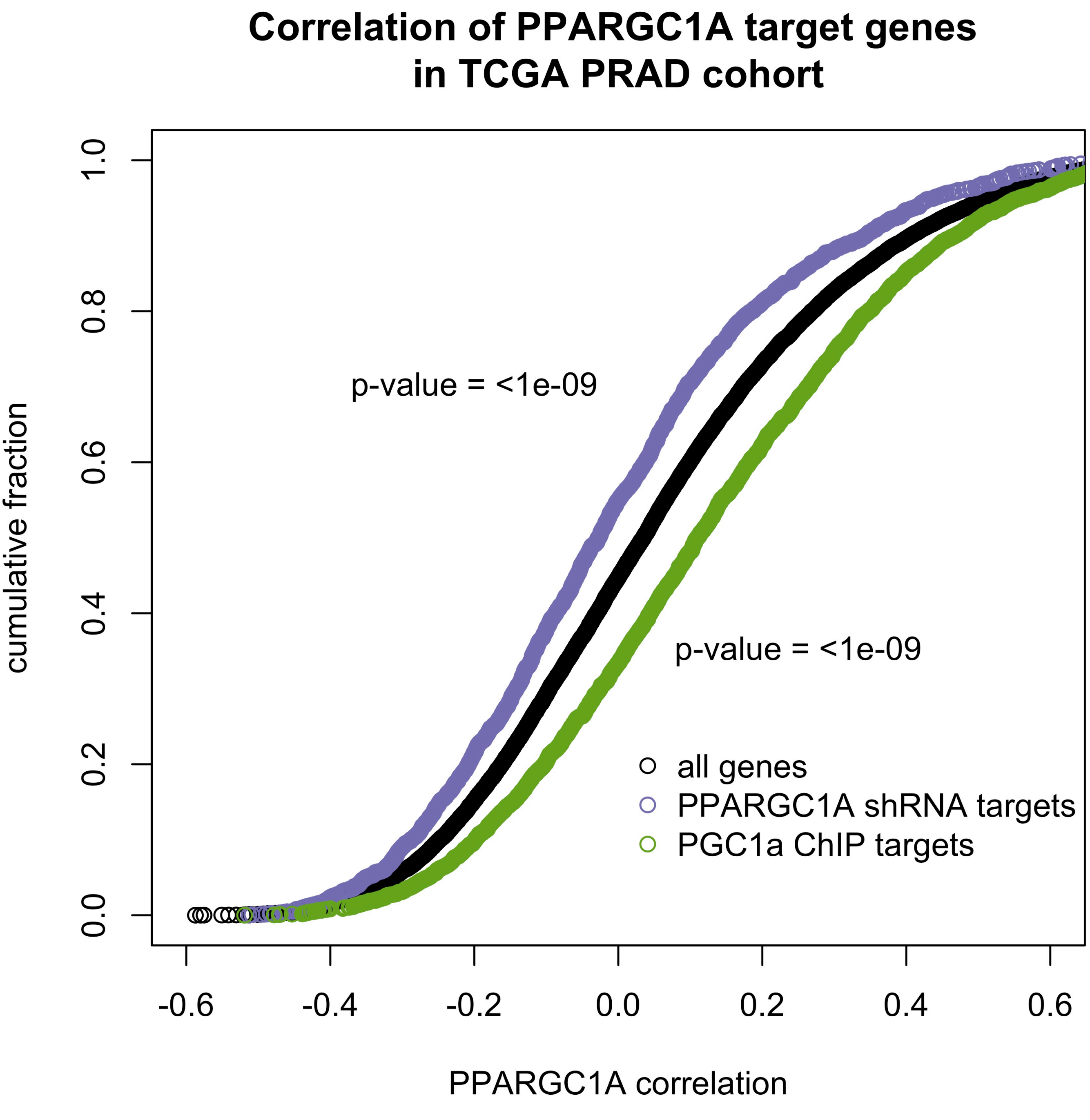

Supplementary Figure 6

Cell_Line	V1
NCIH660_PROSTATE	0.47613
VCAP_PROSTATE	–3.426818
MDAPCA2B_PROSTATE	–0.9270735
DU145_PROSTATE	–5.0276
LNCAPCLONEFGC_PROSTATE	–3.660012
X22RV1_PROSTATE	–4.357137
PC3_PROSTATE	–9.887421
PRECLH_PROSTATE	–4.978315

Supplementary Figure 7
